# Chain relationship between cumulative ecological risk and physical activity pattern on college students mediated by exercise atmosphere and self-control

**DOI:** 10.1038/s41598-025-19836-4

**Published:** 2025-10-14

**Authors:** Tianyi Chen, Yue Gao, Zixia Bu, Xu Du, Mo Sha, Jiahui Liu, Fuqiang Dong, Jizhao Li

**Affiliations:** 1 Beijing Sport University, Beijing, China; 2 College of P.E and Sports, Beijing Normal University, Beijing, China; 3 School of International Chinese Language Education, Beijing Normal University, Beijing, China; 4 Physical Education Department, Beijing University of Posts and Telecommunications, Beijing, China; 5 Department of Physical Education, Communication University of China, Beijing, China; 6 Department of Physical Education, Baoding University, Hebei, China; 7 College of Physical Education, Minzu University of China, Beijing, China

**Keywords:** College student, University physical education, Cumulative ecological risk, Exercise atmosphere, Self-control, Human behaviour, Psychology, Patient education, Quality of life, Rehabilitation, Lifestyle modification, Health care, Disease prevention, Preventive medicine

## Abstract

To investigate the effect of cumulative ecological risk (CER) on college students’ physical activity patterns (PAP) and to analyze the mediating effect of exercise atmosphere (EA) and self-control (SC), with the aim of promoting the convergence of university physical education with community sports and helping college students to form a healthy physical lifestyle. A total of 966 college students were selected from schools in Beijing, China, and a cross-sectional survey of cumulative ecological risk, physical activity patterns, exercise atmosphere, and self-control was conducted using four scales. Differences, correlations and mediated models were analyzed using ANOVA, Pearson and structural equation modelling (SEM). CER negatively predicted PAP (β=-0.293, *P* < 0.01), SC (β=-0.523, *P* < 0.01), and EA (β=-0.352, *P* < 0.01). EA positively predicted SC (β = 0.358, *P* < 0.01) and PAP (β = 0.084, *P* < 0.01), while SC positively predicted PAP (β = 0.256, *P* < 0.01). Mediation analysis using the Bootstrap method indicated that CER affected PAP both directly and indirectly through three significant paths: Direct effect: CER→PAP (effect size = 0.636). Indirect effects: CER→EA→PAP, CER→SC→PAP, and CER→EA→SC→PAP (combined effect size = 0.364). This also indicates that CER can directly reduce PAP among college students, while effectively enhancing EA and SC, thereby mitigating the negative effects of CER. (1) CER directly affects college students’ sport lifestyles, and family and school risks account for the largest proportion of the total, so resolving an individual’s relationship with family and school is an important way to improve sport participation. (2) Both EA and SC positively predict PAP and mitigate the negative effects of CER on it, so increasing the levels of both is also an effective way to improve individuals’ sport experiences.

## Introduction

A sporting lifestyle is an important part of an individual’s healthy lifestyle and an important way of supporting the overall development of students. Sporting lifestyle refers to the physical and psychological development of the individual through daily sporting activities in accordance with social norms and is an important part of the formation of a healthy lifestyle^1^. In physical education, it is necessary to overcome a certain level of exercise load and intensity, which improves the level of physical and cardiorespiratory fitness of students, brings about adaptive changes in the organism and strengthens the students’ will^2^. According to cumulative ecological risk theory, individual behavior is usually the result of a combination of interpersonal, environmental and individual factors, and the cumulative ecological risk (CER) perspective provides a comprehensive view of the factors that contribute to the emergence of individual behavior^3^. CER refers to the integrated and cumulative socio-ecological environment that adversely affects individuals. According to its theory, an individual’s growth environment is composed of a variety of factors that often overlap and cumulatively affect the individual’s development^4^. Therefore, individual risk factors cannot explain the development of students’ sporting lifestyles and must be included in a comprehensive analysis of cumulative factors such as family, school, society and the individual. If the CER is high, the lack of a good family, school and community fitness environment for college students will hinder the formation of college students’ EA^5^.

Exercise atmosphere (EA) refers to the exercise situation around the individual and the relevant exercise information that is easily accessible, which can effectively guide the individual to exercise for a long time, and a good EA will increase the enthusiasm of college students to exercise, which is conducive to the formation of an active and healthy physical activity pattern (PAP)^6^. PAP refers to the habitual distribution of physical activity over time, including frequency, intensity, and type of activity, which are important determinants of health outcomes^7^. When the CER is small, the school, family and society create an excellent exercise environment for college students, and when college students feel that the number of sports fields and the accessibility of transport meet the expected psychological needs of the individual, it will stimulate the individual’s enthusiasm for exercise^8^. Parents’ positive parenting style and sports beliefs influence their children to form the habit of insisting on exercise, and school, as an important part of students’ daily lives, is conducive to the formation of good PAP among college students through the provision of space and equipment, the implementation of sports activities, and peer support^9^. Based on the herd mentality, positive PAP increases the individual’s recognition of the value of exercise and will lead to a tendency for the individual’s exercise behavior to converge with the exercise habits of the majority of their peers^10^. The development of the individual’s interpersonal relationships and positive adjustment to school life through group exercise will lead to a healthy sporting lifestyle. When PAP is poor, individuals do not feel athletic camaraderie and peer acceptance, may reinforce negative experiences of exercise, may avoid exercise routines, and are not conducive to the development of a good physical activity (PA) lifestyle^11^.

Self-control (SC) refers to an individual’s ability to rationally manage thoughts, emotions and behavior. Previous studies have suggested that people with high SC can think rationally and effectively inhibit negative thoughts, leading to a peaceful state of mind^12^. Individuals with high levels of SC hold more positive beliefs, consciously control possible undesirable behaviors, and tend to adopt positive coping styles, which is an important protective factor for individuals’ emotions and problem behaviors. Insufficient levels of SC reduce the brain’s executive function, which makes students vulnerable to negative emotional disorders and problem behaviors. Individuals with higher SC ability will have more and advantages in autonomy, exercise self-efficacy and resistance to interference, which will encourage students to hold positive beliefs about physical exercise, improve their ability to focus attention and take the initiative to avoid the occurrence of undesirable behaviors^13^. Individuals with poor SC ability, on the other hand, are unable to control their emotions and behavior when encountering unfavorable environments during exercise, which is manifested in weaker quality of will, lack of organizational and managerial skills for the given exercise task, and difficulty in concentrating on the exercise task^14^.

**Fig. 1:**
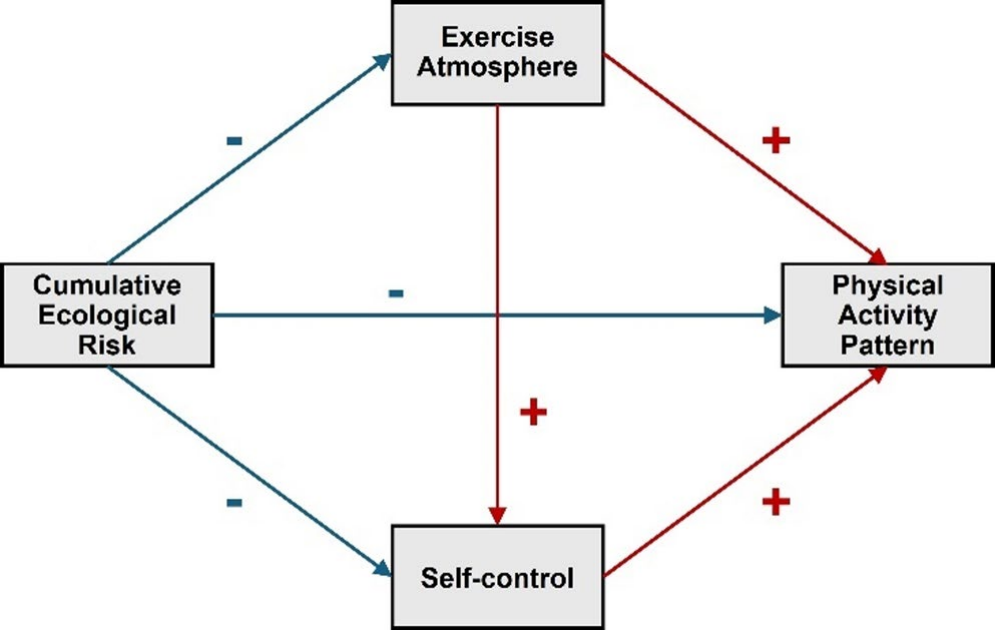
A hypothetical framework based on four variables. Note: Red indicates a positive predictive effect, while blue indicates a negative predictive effect.

According to motivational frameworks such as Self‑Determination Theory and Social Cognitive Theory, both PAP and SC serve as key mediators in the relationship between CER and students’ EA. SDT posits that satisfaction of the basic psychological needs for autonomy, competence, and relatedness enhances intrinsic motivation, which in turn facilitates persistent PA^15^. In this context, a positive PAP—reflecting consistent, self‑regulated engagement with physical activity—creates a supportive environment that nurtures both individual autonomy and peer‑based encouragement, thereby strengthening SC (e.g., autonomy support and self‑regulation skill development)^16^. Meanwhile, SCT suggests that self‑efficacy (a key aspect of SC) and environmental influences operate in a reciprocal manner, so that PAP not only reflects behavior but also shapes beliefs and perceived control, which then feedback to further behavior change and social context^17^. Therefore, based on the previous literature, the following hypotheses were formulated in this study: (I) CER can significantly and negatively influence college students’ EA. (II) PAP is a mediating variable between CER and college students’ EA. (III) SC has a mediating effect in the relationship path between CER and college students’ EA. At the same time, the theoretical assumption framework diagram of the four variables is shown in Fig. 1.

## Materials and methods

### Participants

A random cluster sampling method was employed to select 966 undergraduate students from the classes in each college of the university in Beijing. The inclusion criteria for the subjects were as follows: (1) The study population consisted of currently enrolled undergraduate students, exclusively those pursuing full-time studies. (2) The subjects were required to demonstrate clear consciousness and normal behavioral ability, the capacity to complete the questionnaire, and the absence of an intellectual disability and a history of mental illness. (3) The subjects participated in the survey work of this study on a voluntary basis and did not indicate any negative phenomena. (4) The informed consent to participate in the survey was provided by the counsellor (classroom teacher) in the form of a signed consent document, after which the subject proceeded to complete the questionnaire. (5) The subjects were not Made aware of the necessity for completion of the questionnaire and displayed no difficulty in doing so. In the end, 941 scales with valid results were obtained, and the validity rate of the questionnaires was 87.45%. Demographic information and scale collection results are detailed in Table 1.

**Table 1:** Participants’ physical characteristics.

Parameter		Value
Male		579
Female		387
First year of college		260
Second year of college		308
Third year of college		227
Fourth year in college		171
Type of specialization	Science student	434
	Humanities student	532
Age (yr)		20.36 ± 1.78
Body mass (kg)		75.29 ± 7.52
Height (m)		1.74 ± 4.69
BMI (kg/m2)		24.91 ± 1.82
Screen usage time (h/day)		8.17 ± 2.45
Sedentary behavior (h/day)		8.47 ± 1.38
Cumulative ecological risk (score)		34.92 ± 11.23
Exercise atmosphere (score)		62.75 ± 13.47
Self-control (score)		54.90 ± 10.01
Physical activity pattern (score)		58.61 ± 14.84
History of exercise	Yes	78%
	No	22%
History of smoking	Yes	19%
	No	81%
History of drinking	Yes	64%
	No	36%
Only child family	Yes	77%
	No	23%

### Procedures

In this study, the parameters of the subjects were measured in terms of CER, EA, SC, and PAP through the use of scales based on a variety of indicators. During the testing process, all indicators were distributed via the SoJum software platform, developed by Tencent Holdings Ltd. Prior to data collection, the research team provided a detailed explanation of the study’s purpose and methodology to the participants. If any questions arose, the participants were invited to discuss them with the research team and, if necessary, with their class teacher. The participants were then asked to provide their consent to proceed with the survey. The scale was completed anonymously, without the inclusion of any personal information such as identity card number or student number, and with the exclusion of invalid responses, questionnaires completed in less than 200 s, 10 consecutive questionnaires with the same option, and questionnaires with high homogeneity. The procedures were conducted in accordance with the National Institutes of Health Guidelines and were approved by the Ethics Committee of Human Experimentation of Beijing Normal University (Authorization No. TY20220905). A statement to confirm that all methods were carried out in accordance with relevant guidelines and regulations. Informed consent form was obtained from all subjects.

### Measurements

#### Cumulative ecological risk

Li’s CER questionnaire, revised in 2016, was selected. In total, it covers 5 dimensions of family risk, peer risk, school risk, community risk and other risk, including 46 question items^18^. It is scored on a scale of ‘1’ (very non-compliant) to ‘5’ (very compliant), with the total score representing the level of CER. As CER is a negative variable, this paper provides a reverse scoring process for the relevant question items and the total score is used as the result of CER. The Cronbach’s alpha of the scale is 0.883, indicating a high reliability of the scale.

#### Exercise atmosphere survey

This study used Liu’s revised exercise atmosphere scale^19^. The scale consists of 17 questions and 5 dimensions, including interpersonal association, natural association, information access, interpersonal barriers and conditional barriers. The scale was scored on a 5-point Likert scale from ‘1’ (strongly disagree) to ‘5’ (strongly agree), and the total score indicated the strengths and weaknesses of the EA. The Cronbach’s alpha of the scale in this study reached 0.775, indicating a high reliability of the scale.

#### Physical activity pattern

Participants’ PAP was assessed using the Physical Activity Pattern Scale for College Students (PAP) revised by Wang, which includes 6 dimensions with 30 questions on healthy, challenging, pleasurable, athletic, learning and social^20^. A 5-point Likert scale was used, ranging from ‘1’ (not at all true) to ‘5’ (completely true), and the total score represented the participant’s PA lifestyle. The Cronbach’s alpha of the scale was 0.812, indicating a high reliability of the scale.

#### Self-control level

This study used the Self-Control Scale developed by Tangney in 2004 and later revised by Tan^21^. Its 19 items were selected from the original 36 items. A 5-point scale is used, with scores ranging from 1 to 5, corresponding to ‘not at all compliant’ to ‘fully compliant’, with items 1, 5, 11 and 14 scored positively and the remaining items scored negatively, with the higher the total score, the stronger the SC. The scale Has 3 dimensions, including emotion, thinking and behavior. The reliability of the scale is high, with a Cronbach’s alpha of 0.879.

### Statistical analysis

In this study, we employed a range of analytical techniques, including difference comparison, correlation analysis and mediation chain modelling, to examine the indicators in question. Firstly, SPSS 21.0 was employed to assess the normality of each indicator individually, with descriptive statistics subsequently conducted following the determination of the data type. Given that the primary variables in this study were categorized as light, medium and high levels of PA, the analysis of variance (ANOVA) was utilized, with the P, F values and 95% CI reported. Data that did not align with the hypotheses were corrected using the Greenhouse-Geisser Method. In order to explore the correlation and effect size among variables, Pearson’s test was employed for the analysis of the results, with r and P values subsequently reported. Following the screening of the correlation results, the valid variables were incorporated into structural equation modelling (SEM) through AMOS 24.0 for the purpose of conducting mediated chain model analysis and fitting, to explore the path characteristics among variables. Following standardization of the results, model 6 of the SPSS process canonical macro program plug-in was employed for the purpose of conducting a chained mediation effect test and estimation of confidence intervals. Subsequently, the significance of each path was evaluated through the application of the bootstrap method. Subsequently, the Bonferroni test was employed for post hoc analysis of all indicators, with a significance threshold of *P* < 0.05.

## Results

### Descriptive statistics and gender differences results

To provide a clearer understanding of the overall distribution of key variables and potential gender-based variations, this section presents the descriptive statistics and gender comparisons of CER, EA, PAP, and SC. These analyses lay the groundwork for subsequent modeling and help identify whether gender differences may influence the relationships among core variables.

In terms of CER, the total score was higher among females (5.62 ± 3.36) than males (4.61 ± 3.27), with the difference reaching statistical significance (t = 6.64, *P*<0.01). Specifically, females reported significantly higher levels of family, peer, school, and other risk (all *P*<0.01), whereas no significant gender difference was observed in community risk (*P* = 0.34).

Regarding EA, males scored significantly higher on the total scale (30.15 ± 24.28) compared to females (15.93 ± 16.87, t = 9.18, *P*<0.01). Subscale analysis showed males perceived greater natural association, information access, and interpersonal barriers (all *P*<0.01), while interpersonal association and conditional barriers did not differ significantly by gender (*P*>0.05).

In terms of PAP, males again showed significantly higher total scores (5.69 ± 3.02) than females (4.26 ± 3.01, t = 7.01, *P*<0.01). Subcomponents including challenging, pleasurable, athletic, and learning patterns all favored male participants with significant differences (all *P*<0.01). No significant gender differences were observed in the healthy and social subdimensions (*P*>0.05).

Finally, for SC, female students reported significantly higher total scores (5.28 ± 3.16) than males (4.37 ± 3.02, t = 3.79, *P*<0.01). Among subdimensions, emotional and behavioral control were both significantly higher among females (*P*<0.05), while thinking control did not show a significant difference (*P* = 0.48). The details have shown in Table 2.

**Table 2:** Gender differences in each variable and descriptive statistics.

Parameters		Total	Male	Female	t	*P*
Cumulative ecological risk (score)	Total score	4.86 ± 3.46	4.61 ± 3.27	5.617 ± 3.36	6.64	<0.01**
	Family risk,	0.94 ± 0.66	0.82 ± 0.69	1.64 ± 0.68	7.01	<0.01**
	Peer risk	0.91 ± 0.77	0.81 ± 0.73	1.08 ± 0.72	6.38	<0.01**
	School risk	1.03 ± 0.86	0.87 ± 0.75	1.25 ± 0.89	7.15	<0.01**
	Community risk	0.72 ± 089	0.59 ± 0.63	0.63 ± 0.64	1.71	0.34
	Other risk	0.88 ± 0.82	0.65 ± 0.84	1.06 ± 0.89	6.39	<0.01**
Exercise atmosphere (score)	Total score	24.46 ± 22.71	30.15 ± 24.28	15.93 ± 16.87	9.18	<0.01**
	Interpersonal association	2.46 ± 0.80	0.43 ± 0.89	0.51 ± 0.82	1.67	0.73
	Natural association	2.64 ± 1.15	2.91 ± 1.13	2.2 5 ± 1. 06	5.04	<0.01**
	Information access	3.35 ± 1.23	3.60 ± 1.19	2.98 ± 1.20	8.76	<0.01**
	Interpersonal barriers	3.24 ± 1.09	3.44 ± 1.07	2.93 ± 1.05	4.28	<0.01**
	Conditional barriers	2.12 ± 0.49	0.11 ± 0.48	0.14 ± 0.51	1.31	0.33
Physical activity pattern (score)	Total score	4.91 ± 0.93	5.69 ± 3.02	4.26 ± 3.01	7.01	<0.01**
	Healthy	0.95 ± 0.79	1.06 ± 0.65	0.79 ± 0.65	1.03	0.42
	Challenging	0.12 ± 0.63	0.14 ± 0.51	0.11 ± 0.48	6.5	<0.01**
	Pleasurable	0.83 ± 0.90	1.01 ± 0.88	0.69 ± 0.80	5.94	<0.01**
	Athletic	1.54 ± 0.76	1.79 ± 1.15	1.34 ± 1.14	4.39	<0.01**
	Learning	0.37 ± 0.29	0.48 ± 0.22	0.21 ± 0.14	3.11	<0.01**
	Social	0.31 ± 0.66	0.32 ± 0.11	0.29 ± 0.15	1.38	0.17
Self-control (score)	Total score	4.96 ± 2.82	4.37 ± 3.02	5.28 ± 3.16	3.79	<0.01**
	Emotion	1.98 ± 7.84	1.86 ± 0.65	2.06 ± 0.68	2.57	<0.01**
	Thinking	1.15 ± 0.52	1.12 ± 0.45	1.16 ± 0.55	1.69	0.48
	Behavior	1.85 ± 0.97	1.72 ± 0.83	1.91 ± 0.87	2.05	<0.04*

Note: All variables have been standardized, *=*P* < 0.05, **=*P* < 0.01.

### Common method deviation test results

Prior to the formal distribution of questionnaires to college students, participants were provided with rigorous operational training to comprehensively mitigate the impact of common method bias. This was achieved through the implementation of several strategies, including the use of standardized instructions, strict control of filling time, mixed distribution of positive and negative questions, and the control of demographic variables. These techniques were drawn from the existing literature on the subject Matter. Concurrently, the Harman one-way test was employed to incorporate all the items of CER, PAP, SC and College Student Physical Lifestyle Scale into the exploratory factor analysis. The results demonstrated that a total of 16 factors exhibited eigenvalues exceeding 1, with the initial factor accounting for 26.72% of the variance, which was considerably lower than the established reference standard of 40%. Therefore, the present results were validated.

### Correlation test results between variables

**Fig. 2:**
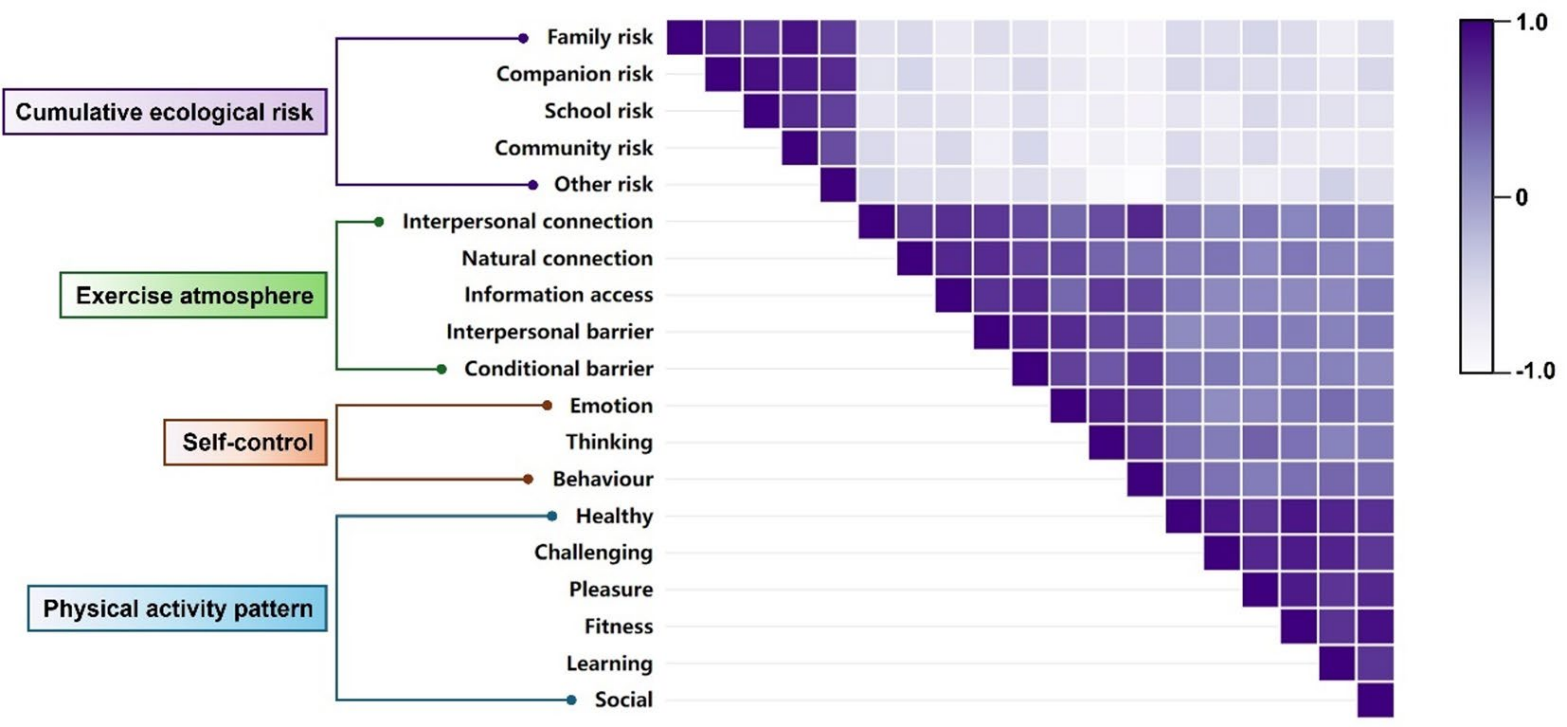
Correlation heatmap between individual variables.

The correlation results showed that CER was significantly negatively correlated with PAP, SC, and EA after controlling for gender, grade, and major by Pearson’s correlation test (r=[−0.761, −0.235], *P* < 0.05); PAP was significantly positively correlated with college students’ SC and EA (r=[0.237, 0.921], *P* < 0.05); and there was a significant positive correlation between SC and college students’ EA (r=[0.221, 0.0.830], *P* < 0.05). This result also indicates that more than 90% of the main variables and sub-indicators are significantly correlated with each other, and further mediation effect tests can be conducted^22^. Detailed information can be found in Table 3; Fig. 2.

**Table 3:** Correlation results between different variables.

Parameter	x¯±s	Sub-parameter																			
			Family risk	Companion risk	School risk	Community risk	Other risk	Interpersonal connection	Natural connection	Information access	Interpersonal barrier	Conditional barrier	Emotion	Thinking	Behaviour	Healthy	Challenging	Pleasure	Fitness	Learning	Social
Cumulative ecological risk	151.66 ± 27.15	Family risk	1.00																		
		Companion risk	0.82	1.00																	
		School risk	0.73	0.92	1.00																
		Community risk	0.89	0.85	0.77	1.00															
		Other risk	0.68	0.77	0.65	0.58	1.00														
Exercise atmosphere	61.37 ± 10.36	Interpersonal connection	−0.40	−0.42	−0.44	−0.32	−0.29	1.00													
		Natural connection	−0.33	−0.30	−0.37	−0.45	−0.36	0.69	1.00												
		Information access	−0.50	−0.48	−0.40	−0.32	−0.35	0.75	0.80	1.00											
		Interpersonal barrier	−0.35	−0.43	−0.50	−0.57	−0.49	0.70	0.77	0.73	1.00										
		Conditional barrier	−0.41	−0.32	−0.37	−0.31	−0.36	0.61	0.64	0.79	0.87	1.00									
Self-control	50.61 ± 14.28	Emotion	−0.57	−0.49	−0.57	−0.64	−0.48	0.45	0.62	0.44	0.76	0.64	1.00								
		Thinking	−0.64	−0.56	−0.55	−0.59	−0.69	0.59	0.46	0.69	0.62	0.53	0.83	1.00							
		Behavior	−0.62	−0.56	−0.63	−0.65	−0.76	0.78	0.39	0.61	0.55	0.70	0.69	0.76	1.00						
Physical activity pattern	58.73 ± 13.23	Healthy	−0.34	−0.31	−0.44	−0.34	−0.32	0.39	0.33	0.37	0.24	0.39	0.37	0.40	0.44	1.00					
		Challenging	−0.37	−0.33	−0.55	−0.46	−0.43	0.27	0.38	0.24	0.28	0.36	0.22	0.33	0.39	0.88	1.00				
		Pleasure	−0.30	−0.36	−0.32	−0.34	−0.53	0.37	0.25	0.25	0.36	0.27	0.26	0.48	0.32	0.71	0.79	1.00			
		Fitness	−0.36	−0.35	−0.38	−0.48	−0.47	0.28	0.36	0.24	0.32	0.29	0.35	0.39	0.40	0.88	0.85	0.85	1.00		
		Learning	−0.55	−0.45	−0.41	−0.51	−0.26	0.34	0.29	0.25	0.29	0.30	0.42	0.29	0.45	0.80	0.80	0.71	0.72	1.00	
		Social	−0.40	−0.31	−0.42	−0.49	−0.39	0.25	0.27	0.34	0.35	0.24	0.34	0.34	0.41	0.73	0.69	0.78	0.91	0.71	1

### Mediation effect test between variables

In this study, SEM analyses were conducted using AMOS 24.0 to test the path effect values and goodness of fit between the variables. SEM is a large-sample data estimation method, and the sample size cannot be too large or too small. In order to ensure that the sample size is sufficient for accurate results, the method proposed by MacCallum, Browne & Sugawara was used to calculate the sample validity before analyzing the model^23^. Assuming a model check value of 0.8, H0 = 0.05 and H1 = 0.08 in the RMSEA hypothesis with a predicted degree of freedom of 100, respectively, confirmed that the sample size included in this study met the basic requirements. In order to make the model more streamlined, the results of the sub-dimensions of all variables were over-combined to observe the predictive relationship and fit of the main variable CER with the three dependent variables. The fit indices of the model are: χ2/df = 1.327, *P* < 0.001, GFI = 0.902, NFI = 0.910, CFI = 0.916, TLI = 0.935, RMSEA = 0.052, which meets the requirements of psychometrics. The detail is shown in Table 4.

**Table 4:** Fitting degree of original hypothesized model.

	X2(DF)	X2(DF)	GFI	AGFI	SRMR	RMSEA	NFI	IFI	TLI (NNFI)	CFI
Fitting value	121.043	1.327	0.902	0.928	0.059	0.052	0.910	0.921	0.935	0.916
Byrne value		1 < x2/DF < 3	> 0.90	> 0.90	< 0.08	< 0.08	> 0.90	> 0.90	> 0.90	> 0.90

**Fig. 3:**
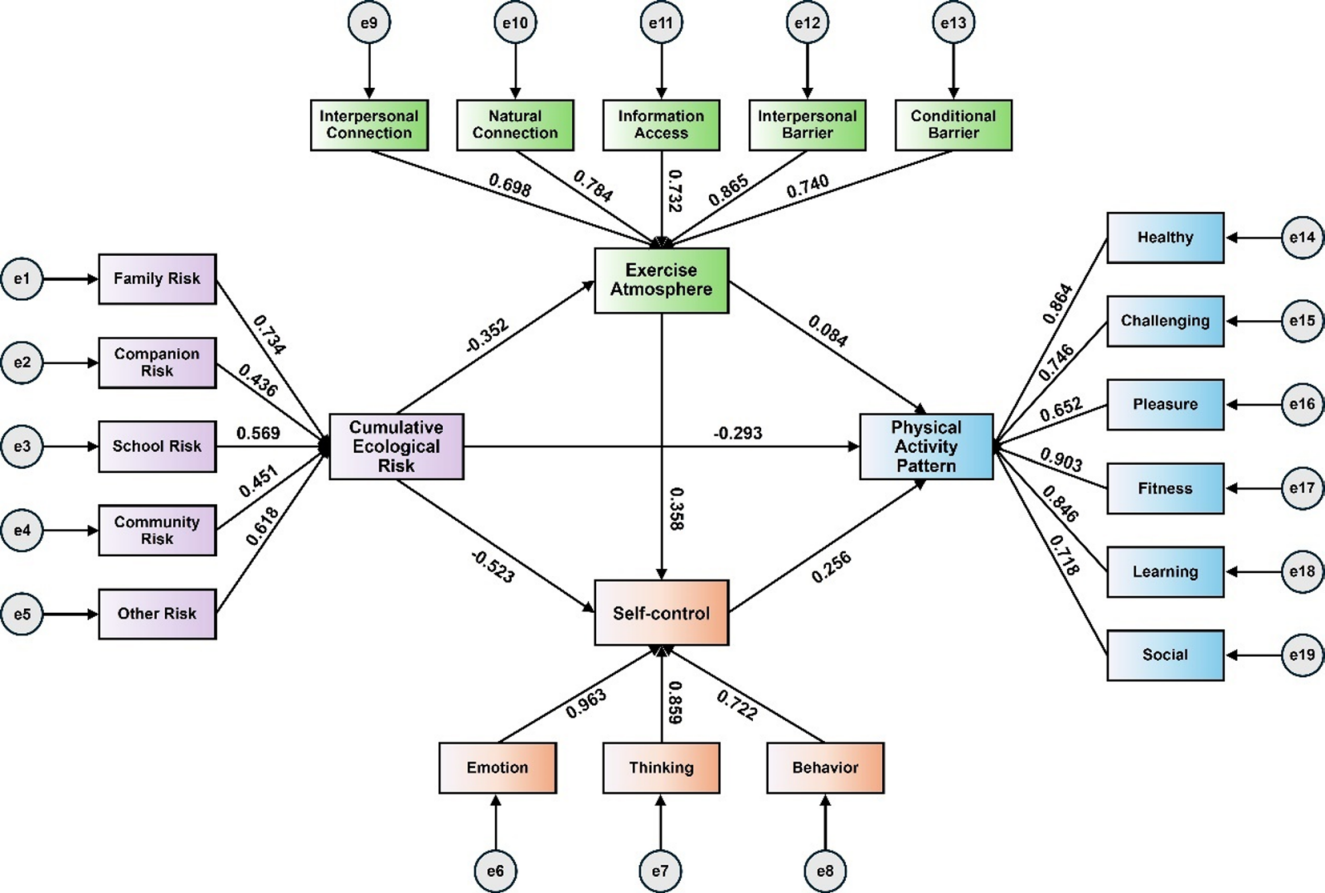
Chained intermediary model and path between variables. Note: CER = cumulative ecological risk, PAP = physical activity patterns, EA = exercise atmosphere, SC = self-control, (+) is positive predictive effect, (-) is negative predictive effect.

The mediating effect of PAP and SC in CER in influencing college students’ physical education lifestyle pathways was tested. In this paper, the demographic variables of college students were strictly controlled, and other variables were standardized. Regression analyses showed that CER significantly negatively predicted PAP (β=−0.293, *P* < 0.01), EA (β=−0.352, *P* < 0.01), and highly negatively predicted SC levels (β=−0.523, *P* < 0.01). EA was able to have a significant positive effect on SC (β = 0.358, *P* < 0.01), and PAP (β = 0.084, *P* < 0.01). There is a positive predictive effect of SC on PAP (β = 0.256, *P* < 0.01). Based on the above results, the chain mediation model was constructed, and the path coefficient results showed that CER had the greatest degree of influence on college students‘ EAs, SC had the second greatest influence on college students’ EAs, and PAP had a lesser influence effect. Detailed information is shown in Fig. 3; Table 5.

**Table 5:** Chained intermediation model and regression analysis based on PA improving IA.

Variable	Equation 1 (Dependent variable: PAP)			Equation 2 (Dependent variable: SC)			Equation 3 (Dependent variable: EA)		
	β	S.E.	t	β	S.E.	t	β	S.E.	t
CER	−0.293	0.027	−3.652**	−0.523	0.031	−6.478**	−0.352	0.058	−5.021**
EA	0.084	0.042	2.426**	0.358	0.026	7.324**			
SC	0.256	0.020	5.548**						
R^2^		0.201			0.68			0.36	
F		28.56**			81.13**			30.54**	

Note: *=*P*<0.05, **=*P*<0.01.

### Results of path fit analysis between variables

In this study, the mediating effect of each indirect path effect was examined, and its confidence interval was estimated by Bootstrap procedure test with repeated sampling 5000 times. The test results show that all variables can constitute 3 indirect pathways and Bootstrap 95% confidence intervals for all pathway values do not contain 0, which indicates that the mediation effect of all pathways reaches a significant level. In this model, the mediating effect consists of the indirect effects generated by 4 mediating paths, of which, the total standardized indirect effect value is −0.162, 95%CI=[−0.3390, −0.1684], which accounts for 36.48% of the total effect. The results of the indirect effects of the 3 mediating paths are as follows: (1) The effect value of the path that CER→EA→PAP had an effect value of −0.024, 95%CI=[−0.0608, −0.0114], accounting for 5.41% of the total effect value. (2) The ‘CER→SC→PAP’ pathway had an effect value of −0.029, 95%CI=[−0.1615, −0.0659], accounting for 22.13% of the total effect value. (3) The effect value of ‘PA→EA→SC→PAP’ was − 0.031, 95%CI=[−0.0356, −0.0143], accounting for 8.96% of the total effect value. In addition, the total standardized direct effect value of CER on PAP was − 0.266, 95%CI=[−0.3264, −0.1802]. The specific structural equation model and path parameters are shown in Table 6.

**Table 6:** Bootstrap analysis of the mediating effect test.

Type of effect	Pathway	Effect value	Boot 95%CI		Effect value ratio
			Upper limit	Lower limit	
Direct effect	CER→PAP	−0.266	−0.1802	−0.3264	0.636
Indirect effect	CER→EA→PAP	−0.024	−0.0114	−0.0608	0.054
	CER→SC→PAP	−0.029	−0.0659	−0.1615	0.221
	PA→EA→SC→PAP	−0.031	−0.0143	−0.0356	0.089
Total indirect effect		−0.162	−0.1684	−0.3390	0.364
Total effect		−0.178			1

Note: CER: cumulative ecological risk, EA: exercise atmosphere, SC: self-control, PAP: physical activity pattern.

## Discussion

PAP is an important part of students’ healthy lifestyles and refers to the way in which individuals promote their physiological and psychological health through daily PA. A good PAP increases students’ motivation to exercise, enhances their recognition of the value of exercise, and strengthens their motivation to continue exercising^24^. In addition, parents’ beliefs about sport and school sport resources positively influence students’ sporting behavior. In line with the herd mentality, positive PAP encourages students to imitate the sporting habits of their peers, increases social support and sporting friendships, and fosters the development of a healthy sporting lifestyle^25^. In our study, both PAP and SC served as mediators between CER and students’ EA. Meanwhile, there was a significant relationship between PAP and students’ SC. Positive PAP creates an environment in which individuals monitor and support each other, which effectively improves individual SC. Therefore, this study investigates whether PAP and SC can mitigate the negative effects of CER on PAP.

### The negative impact of cumulative ecological risk on college students’ sporting lifestyle and improvement measures

The first finding of this study is that CER negatively influences college students’ sporting lifestyle, a process shaped by multiple environmental and individual factors such as family, peers, community, and SC. When the CER factor is small, the school, family, and society generally provide a favorable sports environment and sufficient facilities, which facilitate students’ participation in PA^26^. The scientific planning of community facilities ensures convenience for exercise, schools promote activities and clubs through various systems, and parents’ strong awareness of exercise fosters positive imitation in students’ EAs. Parents not only provide equipment but also exercise with their children and supervise health behaviors, which promotes the development of healthy lifestyles^27^. Peer support also plays an important role, influencing students’ choice and adherence to sporting programs, while school leaders’ emphasis on PA, the activity of clubs, and the overall culture provide organizational support for forming stable exercise habits^28^. Furthermore, the accessibility of school and community facilities, together with a supportive green environment, attract students to actively engage in outdoor PA^29^. By contrast, when the CER factor is high, the absence of these resources exposes individuals to ecological adversity. Insufficient SC and coping efficacy make it harder to adopt healthy lifestyles^30^. Under such conditions, college students lack adequate sports facilities, well-maintained equipment, and effective planning or management of spaces, while accessibility to PA opportunities is also restricted. These barriers hinder the formation of a PA lifestyle and reinforce the negative impact of ecological risk^31^.

### The moderating and mediating effects of exercise atmosphere on the relationship between cumulative ecological risk and physical activity patterns

The second finding of this study was that PAP partially mediated the pathway between CER and college students’ EA. When college students are in an environment with less CER, a good exercise environment and harmonious interpersonal support constitute important conditions for the formation of their EAs^32^. Regarding the exercise environment, schools increase investment in sports funding, optimize the supply of school sports resources, and place simple and practical sports equipment in living areas, libraries, dormitories, classrooms, and other spaces^33^. Schools make full use of existing sports grounds and facilities, strengthen their planning, scientifically plan gyms in dormitory and residential areas, extend the opening hours of existing gyms, and assign sports instructors to guide students, thereby improving the convenience of fitness activities. The school’s official website and various social platforms promote the value of sports and health, recommend sports and health knowledge and skills, and guide students to actively integrate sports into daily life^34^. The community sports environment and services also play an important role in shaping students’ EAs. The provision and accessibility of community sports facilities and equipment, the development of sports clubs, the promotion of health knowledge through the media, regular sports and health forums organized by the community, and community fun activities and family sports gatherings create a supportive sports environment, attracting students to maintain regular exercise and forming healthy lifestyles^35^. A good sports environment fosters a favorable PAP for students. Within this environment, parents encourage and continuously motivate students to adhere to physical exercise to improve their health, while physical education teachers focus on cultivating students’ sports literacy and values, which contributes to the formation of a healthy lifestyle^36^.

### The mitigating effect of exercise atmosphere on cumulative ecological risk and the direction of school reform

The third finding of this study is that when college students are in environments with low CER, schools and communities provide safer, more convenient, and more accessible facilities, which increase supportive resources for individual exercise. Peers also form a PAP of mutual supervision and encouragement, and students are more likely to engage in healthy PA when they actively seek support from teachers and peers. Such peer interactions help maintain good interpersonal relationships, encourage participation, and reduce undesirable behaviors^37^. According to Bandura’s social learning theory, peer modeling plays an important role in shaping exercise habits^38^. Moreover, supportive environments and peer relationships replenish attentional resources, and exercising with peers creates a mutually supervised context that enhances SC. Individuals with higher SC can focus on the value of PA, maintain persistence, and consciously inhibit impulsive behaviors^39^. They also show better self-management, stronger emotion regulation, and greater alignment with social norms, which facilitates the formation of a healthy sporting lifestyle^40^. In contrast, high CER reflects a lack of supportive resources. Without adequate support from family, school, and peers, students are more likely to seek compensation in other environments, which hinders the development of a healthy lifestyle^41^. Furthermore, when PA environments are poorly organized, and supervision from parents and peers is limited, students’ SC skills are less likely to develop, making them more prone to withdrawal and procrastination when facing challenges, ultimately obstructing the formation of a healthy PA lifestyle^42^.

### Possible reasons for cumulative ecological risk affecting students’ physical activity patterns

For the above phenomenon, this study speculates several possible reasons. During physical exercise, with the reduction of cumulative risks in the family, school, and community, college students demonstrate a higher level of cognition regarding the value and function of PA. Along with gradual improvements and rational layout of sports facilities and fitness equipment, accessible opening hours and well-maintained equipment enhance students’ satisfaction with the exercise environment^42^. Students are influenced by parents’ positive views of sport and by peer support, which encourages relaxation and enjoyment during exercise and promotes engagement with peers who share similar interests. Parents and peers provide supportive resources for individual PA and form a PAP for mutual supervision of shared PA^43^. A favorable PAP offers an emotional and environmental protective field for sustained health behavior. It mobilizes SC, allowing students to adopt a positive and optimistic attitude when facing exercise barriers, consciously eliminate disturbances unrelated to exercise, actively engage in established tasks, and maintain good exercise habits^44^. A supportive environmental and interpersonal atmosphere provides incentives for independent participation, enhances self-control, and helps individuals integrate into collective environments and develop interpersonal relationships through sports. In contrast, when PAP is poor, community and school sports facilities and fitness equipment cannot meet exercise needs^45^. Additionally, without emotional support from parents and peers, students’ exercise self-control decreases, leading to disorganized, individualized exercise behaviors and interruptions in exercise routines, which hinders the development of a healthy physical lifestyle^46^.

In conclusion, CER is significantly negatively correlated with PAP, SC, and college students’ EA. CER directly predicts a negative effect on college students’ EA and indirectly predicts it through the mediating effects of PAP and SC. These findings align with motivational theories emphasizing the role of the environment and self-regulatory capacities in fostering exercise motivation. Notably, Ahmadi proposed a comprehensive classification system of motivational behaviors grounded in Self-Determination Theory, providing a valuable framework for understanding and enhancing motivational interventions aimed at increasing physical activity^47^. Therefore, families, schools, and society should provide college students with a supportive exercise environment and positive interpersonal support, leveraging both online and offline sports activities to encourage physical exercise^48^. Attention should be given to the encouragement and supervision roles of parents, teachers, and peers, while simultaneously enhancing individuals’ self-control to facilitate the development of a healthy exercise atmosphere among college students^49^.

From a practical perspective, universities and communities can enhance students’ physical activity by improving access to sports facilities, creating supportive social environments, and offering structured opportunities for engagement. Faculty, peers, and community programs can provide encouragement and guidance, reinforcing positive exercise behaviors. By combining environmental support, social reinforcement, and strategies that strengthen self-control, these measures can help students develop sustainable exercise habits and promote long-term physical and mental well-being.

### Limitations and suggestions for future research

This study aims to explore the influence of CER on PAP among college students, focusing on the chain mediation effects of EA and SC. Using a structural equation model, the study reveals how social environments, and psychological mechanisms influence exercise behavior patterns, providing theoretical support for health behavior change. Ultimately, the goal is to promote the extension of university physical education toward community-based and lifestyle-oriented approaches. This approach fosters the development of stable and positive physical activity Habits among college students in their daily lives and lays the foundation for cultivating long-term healthy lifestyles. Consequently, a cross-sectional large-sample survey was conducted, encompassing 966 college students in Beijing, with the objective of analyzing the dosage characteristics of EA and SC in improving college students’ PAP with CER as the main variable. Nevertheless, it should be noted that this study is not without limitations.

Firstly, although four indicators are included in this study to facilitate comparison between populations, the experimental design was a cross-sectional survey with no longitudinal follow-up. In examining previous large-sample surveys, it becomes evident that some studies have employed a screening process using the NHANES, SEER, and CHARLS databases, standardizing the indicators through the application of the Weighted Mean Difference (WMD) methodology and subsequently conducting sequential comparisons. This approach offers the advantage of expanding sample size while incorporating parameters from longitudinal interventions. However, it also has the disadvantage of reducing the originality of the original research. Instead of searching previous databases, a cross-sectional sample was included and compared over a period of approximately one year. It is therefore the intention of this study to highlight its originality, although it must be acknowledged that the results may be open to question due to certain limitations.

Secondly, the purpose of this study is to explore the chain relationship between CER, PAP, and SC in order to predict the optimal psychological path for promoting PA among students and guide campus construction reforms. This study employed the Physical Activity Pattern Scale for College Students developed by Wang et al., which focuses on assessing the patterns and subjective experience of physical activity (e.g., regularity, intensity perception, and emotional engagement), rather than documenting specific exercise types such as cycling, walking, or swimming. As a result, detailed information about participants’ specific sports activities was not collected, which constitutes a limitation of the current research. Moreover, given the large sample size, collecting individual-level exercise data with high specificity is methodologically challenging in cross-sectional designs. More precise information about exercise characteristics can be better captured in future longitudinal or interventional studies, where closer behavioral monitoring is feasible.

Finally, the findings of this study should be interpreted with caution due to potential limitations in sampling generalizability. All participants were recruited from universities in Beijing, which may introduce regional or cultural biases affecting students’ physical activity patterns, self-control capacities, and perceptions of exercise atmosphere. These contextual factors could limit the applicability of the results to other populations. Additionally, the cross-sectional nature of the study restricts the ability to draw causal conclusions. Future research would benefit from longitudinal tracking or experimental designs to clarify the temporal relationships among CER, SC, EA, and PAP. Incorporating targeted interventions aimed at enhancing SC or optimizing EA may also help determine effective strategies for promoting sustainable behavioral change in physical activity engagement.

While the aforementioned issues cannot be overlooked, this study endeavored to minimize the heterogeneity of results caused by the experimental design within the limited experimental conditions by employing techniques such as Bootstrap and Bonferroni. Furthermore, future studies will analyze longitudinal results, PA volume, and gender differences, and re-adjust the core focus of the study. In light of the current findings, efforts will be made to enhance the reliability and rigor of the study. The objective is to provide college students with efficacious exercise prescriptions to improve problematic behaviors and promote mental health.

## Conclusion

(1) CER has a direct impact on college students’ engagement with sports, with family and school-related risks representing the largest proportion of the total. Therefore, addressing the relationship between family and school is an effective strategy for enhancing participation in sports. (2) Both EA and SC have a positive predictive relationship with PAP and serve to mitigate the negative effects of CER on it. Therefore, enhancing the levels of both is also an effective means of improving individuals’ sport experience.

## Data Availability

Data is available on reasonable request. The datasets used and/or analyzed during the current study are available from the corresponding author upon reasonable request.
